# Positive impacts of leisure-time physical activity on cardiorespiratory fitness, co-morbidity level, cardiovascular health and quality of life among midlife adults: a cross-sectional study of a Nigerian population

**DOI:** 10.1186/s13102-023-00622-6

**Published:** 2023-03-06

**Authors:** Fatai Adesina Maruf, Demelum Marylyn Ucheokoye

**Affiliations:** Department of Medical Rehabilitation, Faculty of Health Sciences and Technology, Nnamdi Azikiwe University, Nnewi Campus, Nnewi, Nigeria

**Keywords:** Leisure-time physical activity, Cardiovascular health, Co-morbidity, Quality of life, Midlife adults

## Abstract

**Background:**

Regular physical activity (PA) improves general health and quality of life (QoL) of the general population. It is however not known if leisure-time PA (LTPA) behaviour will reduce co-morbidity and adiposity, and improve cardiorespiratory fitness and QoL in midlife men. This study explored the impacts of regular LTPA behaviour on co-morbidity, adiposity, cardiorespiratory fitness and QoL among male midlife sports club members in a Nigerian population.

**Methods:**

This cross-sectional study involved 174 age-matched male midlife adults: 87 engaging in LTPA (LTPA group) and 87 not engaging in LTPA (non-LTPA group). Information on age, body mass index (BMI), waist circumference (WC), maximal oxygen uptake (VO_2_max)_,_ resting heart rate (RHR), QoL and co-morbidity level was collected using standardized procedures. Data were summarized using mean and standard deviation, and explored using frequency and proportion. Independent t-test, Chi Square and Mann–Whitney U test were employed to determine the impacts of LTPA at 0.05 significance level.

**Results:**

The LTPA group had lower co-morbidity score (p = 0.05) and RHR (p = 0.004), and higher QoL (p = 0.01) and VO_2_max (p = 0.003) than non-LTPA group. While heart disease (χ^2^ = 10.99; p = 0.01) and hypertension (χ^2^ = 15.24; p = 0.004) severity levels were associated with LTPA behaviour, hypertension (p = 0.01) was the only co-morbid condition that had a significantly lower score in the LTPA group than in the non-LTPA group.

**Conclusions:**

Regular LTPA improves cardiovascular health, physical work capacity and QoL in the sample of Nigerian mid-life men. Regular LTPA behaviour is recommended for cardiovascular health promotion, and improved physical work capacity and life satisfaction in midlife men.

**Supplementary Information:**

The online version contains supplementary material available at 10.1186/s13102-023-00622-6.

## Introduction

Chronological ageing, or senescence, is associated with an increased risk of chronic conditions and diseases such as cognitive impairment, cardiovascular disease (CVD), and metabolic syndrome [1, 2]. Due to improved life expectancy, age-related diseases have increased in alarming proportions in recent decades [3]. Studies have suggested that lifestyle factors have a significant impact on how well people age [4–6]. These factors include regular physical activity (PA) behaviour, abstaining from cigarette smoking, and maintaining a healthy body weight [7], and may improve life expectancy and promote successful ageing. Of these lifestyle factors, PA is mostly reported to protect against the deleterious effects of ageing on health and cognition [8].

Studies have demonstrated positive roles of PAs in influencing health status [9–12]. Regular PA improves overall health, and reduces risk of chronic diseases and death [9]. It may also increase average life expectancy through its positive influence on chronic disease development, and impact on secondary ageing through restoration of functional capacity in previously sedentary older adults [13]. Regular PA behaviour promotes life expectancy regardless of body weight status, and is a dominant factor in improving survival and healthy life in adults over the age of 74 yrs [12]. Older adults who regularly and adequately engage in PA have a greater likelihood of aging successfully ten years later [14, 15]. Indeed, regular PA behaviour brings significant health benefits to people of all ages [16]. Thus, to benefit from PA in old age, adequately and broadly, it is only apt to start engaging in PA from a much younger age.

Active lifestyle from middle to older age is associated with longevity [10] and healthy ageing [11, 17], and extends years of active independent living, reduces disability and improves the QoL in older adults [18]. It also improves cardio-respiratory fitness by reducing myocardial oxygen consumption [19]. Indeed, an increase in cardio-respiratory fitness reduces the risk of premature death [20]. Specifically, leisure-time PA (LTPA) is important for successful ageing throughout adult life span [21]. However, reduction in LTPA with age has more to do with physical health limitations than with older age itself [21]. In addition, the benefits of physical health for well-being are due in part to the level of LTPA participation [21]. Thus, individuals can influence their health-related QoL by the way they spend their leisure time. Leisure-time PA refers to all PA behaviours that people engage in voluntarily, in their free disposable time, in a social environment and condition [22]. Compared with other leisure-time behaviours, LTPA can help leisure individuals realize the unification and harmony of bodies and minds, in accordance with the requirements of leisure individuals [23].

Studies have explored QoL and physical function of older people [18, 24, 25], the impacts of PA on mental and psychological status in older adults [6, 26] and the impacts of PA on adiposity indices [27, 28]. However, information on the impacts of regular LTPA on adiposity, co-morbidity level, QoL and cardiorespiratory fitness among midlife adults is scanty. Furthermore, not knowing how PA affects people in midlife may be a missing link in detailed understanding of how the effects of scanty PA behaviour track into older adulthood, and thereby limit understanding of any interventional approach to prevent or manage the impacts on secondary ageing. In addition, the available information on LTPA in the literature comes from non-African populations. Considering the psychosocial differences between African and non-African populations, the findings in previous studies may not easily be extrapolated to an African population. Thus, this study explored the impacts of LTPA on co-morbidity level, cardiorespiratory fitness level, adiposity indices, and QoL among male midlife sports club members in a Nigerian population.

## Methods

This cross-sectional study involved 174 participants (87 LTPA and 87 non-LTPA). It was estimated that the sample size will have 95% power to produce an effect size of 0.5 at 0.05 level of error using G* power 3.0.10. The LTPA group were volunteering midlife male adults who engaged in LTPA at a sports club either in Awka and Onitsha in Anambra State while the non-LTPA group did not. The LTPA group were regular members of the sports club for at least one year, and engaged in LTPA, for at least 30 min, at least two times per week while those in the non-LTPA group were apparently healthy age-matched male adults who did not engage in LTPA. The participants in the two groups gave informed consent to participate in the study.

The Ethics Committee of Faculty of Health Sciences and Technology (Nnamdi Azikiwe University) approved all the procedures employed in this study. Permission of the presidents of each of the sports clubs was obtained, and consent of the participants was also obtained. The LTPA and non-LTPA participants were consecutively recruited from the sports clubs. The LTPA participants were registered members of the sports clubs while non-LTPA participants were non-registered friends of members who accompanied them to the clubs. The LTPA participants had access to the sporting facilities as members while the non-LTPA participants did not. Data were collected on the participants who consented. Information on participant’s age, sex and marital status was collected using a questionnaire. Other pieces of information collected and procedures involved are described as follows:

*Height (m)* This was measured to the nearest 0.01 decimal place using a height meter. The participants stood erect, looked straight ahead and barefooted with the back against the height meter. The measurement was taken on the meter against the vertex of the head.

*Weight (kg)* This was measured to the nearest 0.1 decimal place using a weighing scale. Before measurement, the pointer of the scale was ascertained to be at the zero point. The participant put on minimal clothing and was barefooted. The participant stood erect on the weighing scale, looking straight and relaxed. The reading was taken at the new steady position of the pointer by bending over the scale.

*Body mass index (kg/m*^*2*^*)* The body mass index (BMI) of the participants was calculated from their respective height in meters and weight in kilograms using the formula weight (kg)/height^2^ (m).

*Waist circumference* In carrying out this measurement, the participant’s shirt was raised and if there is any tight belt around the waist it was loosened. The participant stood erect, with feet together and abdomen relaxed, and narrowest part of the trunk was located at the umbilicus. With the participant’s arm lifted up, the measuring tape was evenly placed around the narrowest part of the naked trunk. While in place the tape was pulled lightly without indenting any part of the skin, and then the point on the tape where the measuring end of the tape touched was noted and recorded to the nearest 0.1 cm.

*Aerobic capacity* The VO_2_max was used to assess the aerobic capacity of the participants. The VO_2_ max was estimated using the step bench protocol. Each participant was allowed three minutes rest in relaxed sitting position before mounting the step bench. The Chester Step Bench Protocol is a three-minute sub-maximal aerobic exercise that estimates the VO_2_ max using exercise recovery heart rate. A 12-inch bench was used for this test. The metronome was set at a constant beat frequency of 96 beats per minute for all the participants. This resulted in a cadence of 48 steps per minute for each participant. Participants were instructed to climb up and down the stairs in cadence to the beats of the metronome and to alternate the leading leg at least once or more frequently to avoid fatigue. Once the participant was ready, he was given the command ‘go’ while simultaneously starting the stopwatch. At exactly six minutes, the researcher gave the command ‘stop’, and the subject was asked to sit immediately. The recovery HR was taken at exactly 5 s after the test. This recovery HR was used to estimate the VO_2 max_ (mL/kg/min) using the formula [29]:

$${\text{VO}}_{{{\text{2max}}}} = {111}.{33} - \left( {0.{42} \times {\text{HR}}_{{{\text{recovery}}}} } \right)$$

*Quality of life* Self reported QOL of the participants was assessed using Quality of Life Enjoyment, and Satisfaction Questionnaire Short Form (Q-LES-Q-SF). Q-LES-Q-SF is a 16-item questionnaire derived from the general activities scale of the original 93-item form. It consists of 14 items (assessing satisfaction with physical health, social relations, ability to function in daily life, physical mobility, mood, family relations, sexual drive and interest, ability to perform hobbies, work, leisure activities, and household activities, economic status, living/housing situation, vision and overall well-being) and two additional items (satisfaction with medication and overall life satisfaction but are not included in the overall score) [30]. Each of the 14 items is rated on a five-point scale that indicates the degree of enjoyment or satisfaction experienced during the past week. The total score of all 14 items is computed (ranging from 14 to 70) and is expressed as a percentage (1–100) of the maximum total [30]. The raw total score is transformed to percentage maximum possible score using this formula: (raw total score minus minimum score) divided by (Maximum possible score-minimum score) [30]. Higher scores on the Q-LES-Q-SF indicate greater contentment or satisfaction [30]. The Q-LES-Q-SF has been reported to have an internal consistency of 0.9, a test–retest reliability of 0.93 and a sensitivity of 80% [31].

*Co-morbidity* A self-reported Modified Cumulative Illness Rating Scale (MCIRS) was used to assess the health status and co-morbidity of the participants. The MCIRS has 13 items that cover assessment of health status in different body organs or systems (heart, hypertension, blood vessels, respiratory system, eye/nose/throat/larynx, upper gastrointestine (GI), lower GI, hepatic, renal, other genitourinary, musculo-skeletal-integumentary, neurological, and endocrine-metabolic and psychiatric/behavioral [32]. Each of the 13 items is measured on a scale of 1 to 5 where 1 denotes ‘none’ and 5 denotes ‘extremely severe’ [32]. However, for the purpose of this study, only cardiac, hypertension, endocrine-metabolic (diabetes) and psychiatry/behavioural (mental health) were rated. Co-morbidity score was determined by finding the average of the ratings for the four organ/system areas. High scores indicate high co-morbidity levels and vice versa. The MCIRS has been reported to be valid (r = 0.73 to 0.84) and reliable (interrater: r = 0.78 to 0.81; intrarater: r = 0.80 to 0.89) in a primary care context [33].

### Data analysis

Continuous data were summarized using mean and standard deviation. Categorical data were presented in frequency and percent. Independent t-test was used to compare the examined variables between the participants in LTPA group and those in non-LTPA group. Mann Whitney U test was used to compare the rating for cardiac, hypertension, diabetes and mental health between the two groups. Chi Square was used to determine the association between LTPA status and individual disease severity. Alpha level will be set at 0.05.

## Results

Table 1 shows that 174 participants (87 in LTPA group and 87 in non-LTPA group) were involved in this study. The participants were aged from 40 to 65 years. Participants in the two groups had similar ages, WC and BMI as shown in Table 1. However, Table 1 shows that participants in LTPA group demonstrated significantly lower co-morbidity score (p = 0.05) and resting heart rate (p = 0.004), and better QoL (p = 0.01) and VO_2_max (p = 0.003) than those in non-LTPA group.

**Table 1:** Socio-demographics distributions of participants in the LTPA and non-LTPA participants

Variable	LTPA Mean ± SD	Non-LTPA Mean ± SD	t-value	p value
Age (years)	56.01 ± 7.62	54.25 ± 8.24	1.46	0.15
WC (cm)	91.41 ± 17.27	95.00 ± 16.01	− 1.42	0.16
BMI (kg/m^2^)	31.39 ± 3.78	31.48 ± 5.52	− 0.13	0.90
Co-morbidity	2.71 ± 2.19	3.53 ± 3.08	− 2.02	0.05*
QoL	89.92 ± 9.82	85.32 ± 11.26	2.87	0.01*
VO_2max_ (mL/kg/min)	62.10 ± 5.20	59.66 ± 5.75	− 3.01	0.003*
Heart rate (beats/min)	117.08 ± 12.31	123.02 ± 13.69	2.93	0.004*

*LTPA* leisure-time physical activity, *WC* waist circumference, *BMI* body max index, *QoL* quality of life, *SD* standard deviation

*Statistical significance

Table 2 shows that LTPA status was associated with heart disease severity (χ^2^ = 10.99; p = 0.01) and hypertension severity (χ^2^ = 15.24; p = 0.004). Furthermore, larger number of participants in the LTPA group than in non-LTPA group had no heart disease (LTPA: 88.5% vs non-LTPA: 81.6%), no hypertension (LTPA: 65.5% vs non-LTPA: 52.9%), no diabetes (LTPA: 85.1% vs non-LTPA: 74.7%) and no mental disorder (LTPA: 98.9% vs non-LTPA: 96.6%) (Table 2).

**Table 2:** Distribution of the frequency and percentage of heart diseases, hypertension, diabetes and mental health problems among the participants in the two groups

Co-morbidity	LTPA n (%)	Non-LTPA n (%)	χ^2^	p value
*Heart diseases*				
Normal	77 (88.5)	71 (81.6)	11.0	0.01*
Mild	7 (8.0)	3 (3.4)		
Moderate	3 (3.4)	4 (4.6)		
Severe	–	9 (10.3)		
Extremely severe	–			
*Hypertension*				
Normal	57 (65.5)	46 (52.9)	15.2	0.004*
Mild	18 (20.7)	12 (13.8)		
Moderate	12 (13.8)	17 (19.5)		
Severe	–	10 (11.5)		
Extremely severe	–	2 (2.3)		
*Diabetes*				
Normal	74 (85.1)	65 (74.7)	5.4	0.25
Mild	2 (2.3)	3 (3.4)		
Moderate	9 (10.3)	10 (11.5)		
Severe	2 (2.3)	8 (9.2)		
Extremely severe	–	1 (1.1)		
*Mental disorder*				
Normal	86 (98.9)	84 (96.6)	2.0	0.36
Mild	1 (1.1)	1 (1.1)		
Moderate	–	2 (2.3)		
Severe	–			
Extremely severe	–			

*LTPA* leisure-time physical activity, *n* number of participants

*Statistical significance

Despite the finding that participants in LTPA group had significantly lower co-morbidity score than those in non-LTPA group in Table 1, only the hypertension component of co-morbidity score was observed to have significantly lower rating (p = 0.01) among the participants in LTPA group than those in non-LTPA group (Table 3).

**Table 3:** Mann–Whitney comparing co-morbidity among the participants in the exercising and non-exercising group

Co-morbidity	LTPA (n = 87)	Non-LTPA (n = 87)	Z-value	p value
	Mean rank	Mean rank		
Heart diseases	83.87	91.13	− 1.52	0.13
Hypertension	79.00	96.00	− 2.52	0.01*
Diabetes	82.61	92.39	− 1.83	0.07
Mental disorder	86.49	88.51	− 1.02	0.31

*LTPA* leisure-time physical activity, *n* no of participants

*Statistical significance

## Discussion

This study explored the impacts of LTPA on cardiorespiratory fitness, co-morbidity level, adiposity indices and QoL of midlife adults. The data in this study have shown that the participants in the two groups were similar in age, WC and BMI. However, participants in the LTPA group had significantly lower co-morbidity score and RHR, and better VO_2_max and QoL than those in non-LTPA group. In addition, the data show that LTPA status was associated with heart disease and hypertension severity. Furthermore, hypertension co-morbidity was lower in the LTPA group than in the non-LTPA group.

The finding of impact of LTPA on co-morbidity in this study is similar to the finding in a previous study that LTPA is inversely associated with all-cause mortality in men [34]. Warburton et al. [34] reported that a linear relationship exists between PA and health status, a surrogate index of co-morbidity, such that an increase in PA and fitness will lead to additional improvements in health status. Indeed, beginning a moderate sports activity is associated with a lower risk of death from all-cause and from coronary heart diseases among middle-aged men and older adults [35]. According to da Silva et al. [36], regular LTPA is associated with improved health perception. Similarly, LTPA is associated with a longer life expectancy in individuals with cardio-metabolic multi-morbidity [37]. These benefits from PA behavior are regardless of the type or source [35].

The finding on impact of LTPA on diabetes-related co-morbidity in this study is in contrast to the findings in previous similar studies [38, 39]. The difference in the findings between the current study and the previous ones could be attributed to the design differences in that the previous studies followed up a cohort of participants who engaged in LTPA. In the current study, it is not possible to ascertain that any other diabetes-predisposing factors, such as diet and genetic disposition status, are the same or evenly distributed between the LTPA and non-LTPA groups. The implications of these factors not being similar between the two groups, especially if those factors confer an advantage on non-LTPA group, is that the possible gain by the LTPA group due to their PA participation could be balanced out by the positive factors in non-LTPA group.

In line with a finding in this study, Yu et al. [40] reported no difference in WC and BMI across low-moderate-vigorous categories of LTPA. Indeed, Peterson et al. [41] report that change in LTPA is unrelated to subsequent weight change. In addition, contrary to the findings in this study, previous studies report that LTPA reduces the risk of obesity [42, 43]. This risk is reduced by 5 percentage points with LTPA participation, and by 11 points if LTPA is combined with some work-related PA [43]. The difference in findings between the current and previous studies could be attributed to the difference in study designs.

The finding that hypertension-related co-morbidity was lower in LTPA group than non-LTPA group is similar to the finding that LTPA is associated with lower risk of hypertension among middle-aged men [44]. Similarly, the finding that hypertension occurrence is associated with LTPA status is explained in light of the finding that absence of moderate-vigorous LTPA is associated with an increased risk of hypertension [45]. Indeed, Werneck et al. [46] have reported that engaging in sufficient level of LTPA could attenuate, but not eliminate, the negative influence of obesity on high BP. These findings therefore imply that LTPA could be used to reduce hypertension incidence and severity in midlife men and thereby reduced the risk of cardiac diseases. Indeed, aerobic dance, a form LTPA, has been reported to reduce BP, number of antihypertensive drugs and BP control rate in individual with hypertension [47]. Thus, increasing the knowledge of individuals with hypertension about benefits of PA for their condition will increase their participation in it [48].

In line with findings of association between LTPA and cardiac disease in this study, prior studies have reported that LTPA is inversely associated with risk of CVD [42, 49, 50], especially among sedentary people [49]. Indeed, when leisure time is sedentary, the risk for cardiometabolic diseases increases [51]. LTPA has been reported to reduce carotid artery stiffness [52]. However, this benefit is decreased in those above the age of 65 years, and those with a history of CVD [50]. This association could be mediated by the impacts of LTPA on individual traditional cardiovascular risk factors [53]. These impacts may be attributed to the reduced cardiovascular mortality in moderate- and high-intensity LTPA adherents [49, 54], and the largest reduction is observed with approximately 300 min of walking per week [54], largest intensity of LTPA and after 10 years of LTPA behavior [50]. This amount of LTPA/week translates to approximately 150–300 min/wk of long-term vigorous-intensity LTPA or 300 to 600 min/wk of long-term moderate-intensity LTPA [55].

The finding from this study that there was a higher VO_2_max in participants that engaged in LTPA than those that did not, is similar to those in previous studies [56–58]. In fact, a dose-related increase in VO_2_max from sedentary men, through active men, to endurance-trained men has been observed [59]. In addition, similar to a finding in this study, Yu et al. [40] reported that firefighters who engaged in LTPA had lower resting heart rate than those who didn’t. These findings imply that midlife adult men who engage in LTPA have a higher cardio-respiratory fitness than their counterparts who don’t.

The finding of impact of LTPA on QoL of participants in this study is similar to that of a previous study [60]. However, in contrast to the finding in this study, Tessier et al. [61] reported that the long-term association between LTPA and QoL changes is limited and has little clinical significance, especially for men and for the physical health dimension of QoL. This contrast could be attributed to the different designs employed in the two studies, in which LTPA was self-reported through a questionnaire in Tessier’s et al., study, as against identification of LTPA adherents against their controls in the current study. The recall bias and over-reporting limitations of self-report assessment may be responsible for the contrasting findings between the current study and Tessier’s et al. In addition, Wendel-Vos et al. [62] report that cross-sectional associations are mainly found for physical health dimension of QoL, whereas longitudinal associations are predominantly observed for the mental health dimension. However, a study on effect of aerobic dance, a form of LTPA, on QoL showed larger improvement in physical health, psychological health and environment domains of health-related QoL among participants who engaged in 12-week aerobic exercise than those who didn’t [63]. Generally, PA increased QoL of the physically active men more than inactive men [64]. Nonetheless, Stahl et al. [65] suggest that a social environment is also important for physically active individuals to have good QoL.

The findings that LTPA did not have impact on mental health, and that LTPA status was not associated with mental health severity contradict the finding in a previous study that LTPA had weak positive association with positive mental health [66–68]. The difference in the findings between the two studies could be attributed to self-report mode of determining LTPA in the previous study, which could introduce bias of over- or under-reporting. In addition, the non-LTPA group in the current study may be largely individuals with positive mental health who do not have to benefit significantly from any mental-health-improving intervention. According to Appelqvist-Schimdlechner et al. [69] LTPA and physical fitness may improve mental health, but LTPA, rather than physical fitness, seems to be more essential for positive mental health [69]. Thus, PA strategies aimed at improving mental health for men should provide opportunities, particularly, for LTPA involving social interactions [66]. In addition, low-to-moderate LTPA has been reported to lower the risks for symptoms of depression, burnout, and high stress levels while only moderate-intensity LTPA lowers the risk of anxiety [70, 71]. In addition, high-intensity LTPA may increase the risk of incident depression [70]. Thus, LTPA intervention for mental health has to be symptom-based.

The strength of this study lies in the actual determination of LTPA behaviour status as opposed to use of self-report. The approach adopted in this study is expected to remove group assignment error that characterises the use of self-report due to under- or over-reporting. However, the use of self-report in determining co-morbidity score and disease status in this study may limit interpretation of the finding in this study. Nonetheless, standardized nature of the instrument employed to assess comorbidity is expected to reduce the inherent error associated with the use of such self-report. In addition, the cross-sectional nature of this study does not allow for causal interpretation of the findings.

In conclusion, regular LTPA improves cardiovascular health, physical work capacity and QoL in the sample of Nigerian mid-life men. Regular LTPA behaviour is recommended for cardiovascular health promotion, and improved physical work capacity and life satisfaction in midlife men.

## Supplementary Information

**Additional file 1.** Leisure-time physical activity, cardiovascular risk factors and quality of life.

## Data Availability

All data generated or analysed during this study are included in this published article (and its supplementary information files).

## Abbreviations

LTPA: Leisure-time physical activity
WC: Waist circumference
QoL: Quality of life
PA: Physical activity
BMI: Body mass index
VO_2_max: Maximal oxygen uptake
MCIRS: Modified Cumulative Illness Rating Scale
GI: Gastrointestine
Q-LES-Q-SF: Quality of Life Enjoyment, and Satisfaction Questionnaire Short Form
HR: Heart rate
